# Perceived stigma among patient with pulmonary tuberculosis at public health facilities in southwest Ethiopia: A cross-sectional study

**DOI:** 10.1371/journal.pone.0243433

**Published:** 2020-12-08

**Authors:** Mustefa Mohammedhussein, Mohammedamin Hajure, Jemal Ebrahim Shifa, Tahir Ahmed Hassen

**Affiliations:** 1 Department of Psychiatry, Faculty of Public Health and Medical Sciences, Mettu University, Mettu, Ethiopia; 2 Department of Psychiatry, School of Health Sciences, Mada Walabu University, Shashemene campus, Shashemene, Ethiopia; 3 School of Nursing and Midwifery, College of Health and Medical Sciences, Haramaya University, Harar, Ethiopia; Jamia Hamdard, INDIA

## Abstract

**Objectives:**

Although tuberculosis (TB) related stigma has a significant impact on the diagnosis, patient adherence with treatment, and recovery from the disease, there is limited evidence from Ethiopia regarding perceived stigma among patient with pulmonary tuberculosis (PTB).The purpose of this study was to assess perceived stigma and associated factors among patient with PTB on treatment in southwest Ethiopia.

**Methods:**

Institution-based cross-sectional study was conducted from April to May 2019 among 410 patient with PTB. Data were collected by using the perceived tuberculosis stigma scale. Epi data v3.1 and SPSSv23 were used for data entry and analysis. Multivariable logistic regression models were fitted to identify factors associated with perceived stigma. Results are presented as adjusted odds ratios (AORs) with 95% confidence intervals (CIs).

**Result:**

Prevalence of perceived stigma among patient with Pulmonary tuberculosis was 57.1% (95% CI: 52.2, 61.7). Poor social support (AOR = 2.41; 95% CI: 1.06, 5.48), above a month duration of illness (AOR = 2.48; 95% CI: 1.33, 4.64), high perceived stress (AOR = 1.95; 95% CI:1.09, 3.49), current khat use (AOR = 1.88; 95% CI:1.05, 3.37), and presence of depression (AOR = 8.18; 95% CI:4.40, 15.22) were significantly associated with perceived stigma. Patient with HIV co-infection were 5.67 times (AOR = 5.67; 95% CI: 2.32, 13.87) more likely to have Perceived stigma than their counterparts.

**Conclusion:**

TB related stigma was reported by more than half of the study participant. Stigma reduction measures are needed to lower TB related stigma perceived by the patient, the level of distress associated with it, and to promote the psychological wellbeing of patient with TB.

## Introduction

Tuberculosis(TB) remains a major global health problem despite the availability of diagnostic methods and treatments [1]. Globally an estimated ten million people affected with TB in the year 2018, of which over 95% of the cases and deaths were from developing countries. Ethiopia is one of the 14 countries found in the three lists of high TB burden countries [1].

Stigma is a dynamic process of devaluing that significantly discredits an individual in the senses of others [2]. Disease related stigmas have a significant impact on the effectiveness of disease diagnosis and treatment. Stigma and discrimination towards patient with TB, poor healthcare-seeking behavior, and poor adherence to treatments are among the major challenges for TB control programs particularly in low-income countries [3–8].

The stigma alone remains a major barrier to health care access and has impacts on the physical, social and mental health, and the overall quality of life for individuals with TB. Stigma may lead the patient to often hide the fact of their disease from others and isolate themselves to escape from negative public perceptions [2, 5, 9, 10]. TB related Stigma has a significant impact on the diagnosis, patient adherence with treatment, and the recovery from the disease, and can also affect the care given by the family and health care provider-patient relationship [11, 12].

Studies have been explored that perceived severity of the illness, knowledge about the causation, fear of infection and risk of transmission, co-infection with HIV, being from low socio-economic class, low level of education, depression, gender, and substance use were the factors associated with stigma in patient with TB [3, 13–17].

Several studies revealed that perceived TB stigma may contribute to delayed healthcare seeking, resulting in delays in the diagnosis and initiation of treatment. Delay in TB diagnosis increases the infectiousness of the disease, consequently sustaining transmission within the community. Thus, stigma could be considered as a major obstacle that hinders the success rate of TB control [3, 16, 18, 19].

So far studies have shown that TB is a vastly stigmatized disease and patient with TB had high perceived stigma [15, 18, 20]. In a few studies conducted in Ethiopia, the prevalence of perceived stigma among TB patient ranged from 21.3% to 56% [21–23]. Nevertheless, the magnitude of stigma has been reported to be different across different setting in the country. Addressing the burden of this psychosocial problem in a different setting was vital to design effective intervention strategies to reduce morbidity and mortality associated with the disease. So the current study assessed perceived stigma and its associated factors among patient with PTB at public health facilities in southwest Ethiopia.

## Methods and materials

### Study design and setting

Institution-based cross-sectional study was conducted at public health facilities in the Jimma zone from April- May 2019. Jimma, the zone city is located 352 km far in the southwestern direction from Addis Ababa, the capital city of Ethiopia. During the study period 124 health centers and 7 public hospitals were providing health services for the public in the zone.

### Study population

PTB patient aged ≥18 who had a follow up in selected health facilities during the study period were the study population. Patient who were unable to respond because of the acute exacerbation of the symptoms and those on treatment for less than a month were excluded.

### Sample size and sampling procedures

The sample size for the study was determined by using a single population proportion formula considering a 95% confidence level that falls within a 5% margin of error and taking a proportion of perceived stigma among TB patient 42.4% [23]. Taking a 10% non-response rate into account, the final calculated sample size was 412. Simple random sampling was employed to select 39 health facilities (37 health centers and 2 hospitals) out of 131 public health facilities in Jimma zone. All eligible patient with pulmonary tuberculosis who visited the selected health facilities during the month of April-May 2019 were consecutively interviewed. In order to avoid double-counting, patient attended the selected health facilities more than one time, during the study period, were included only on the first contact.

### Data collection method and instrument

Data were collected through face to face interviews by trained clinical nurses using a translated and pretested questionnaire. A structured questionnaire was used to assess socio-demographic factors. Perceived TB stigma was assessed using the perceived TB stigma scale which was adopted from Somma et al. [21]. The Perceived TB stigma scale consists of 11 items which are rated on a four-point Likert Scale (ranging from 1 = strongly disagree to 4 = strongly agree). Participant were considered to have perceived stigma if scored above or equal to the mean stigma score [21, 24]. This tool has been reported to have good internal consistency in the previous study [21] and had (Cronbach α = 0.84) in the present study.

The perceived level of social support was assessed by Oslo three items social support scale [25]. The tool has demonstrated (Cronbach α = 0.84) in the current study, and scores from 3–8, 9–11, and 12–14 were considered as poor, moderate, and strong social support respectively. The perceived stress level was measured by a ten-item perceived stress scales. A score from 0–15 was considered as low to average perceived stress and a score ≥16 was considered as high perceived stress [26].

Depression was assessed by the depression sub-scale (HAD-D) of the hospital anxiety and depression scale (HADS). This tool has been validated in Ethiopia and its internal consistency was 0.76 for depression subscale. A score ≥ 8 on the HAD-D subscale was considered as a probable case of depression [27]. In the current study, Cronbach α for HAD-D was 0.82.

Data on the HIV co-infection were extracted from the patient chart after informed consent. Duration of illness (the time duration of TB symptoms before the diagnosis of TB) was assessed by a structured questionnaire. Current substance use was assessed by a structured questionnaire and was considered as the use of khat, tobacco, alcohol, and cannabis in the recent three months.

### Study variables

The outcome variable was the perceived stigma, coded as a dichotomous ‘yes/no’ variable. Participant were considered to have perceived stigma (yes) if scored above or equal to the mean stigma score.

The explanatory variables were age (measured in years), sex (male/female), marital status (single, married, divorced, and widowed), educational status (no formal education, primary, secondary, and college and above), religion (Muslim, Orthodox, and Protestant), occupation (unemployed, government-employed, and self-employed), place of residence (urban/rural), family size (less than or equal five /greater than five), perceived social support (poor, moderate and strong), perceived stress level (low to average/high), duration of illness (less than or equal one month/greater than one month), phase of treatment (intensive/continuation), body mass index (underweight/not underweight), category of treatment (new, return after default, and relapse or retreatment), family history of mental illness (yes/no), HIV co-infection (yes/no), and current substance use (yes/no).

### Data quality assurance

The following measures were taken to assure the quality of data. All screening instruments were translated to local language Afaan Oromo and Amharic, and the pretest was performed on 20(5%) of TB patient on follow up at Shenen gibe hospital which was not included in the study. Data collectors were trained on data collection instruments and data handling. Data were regularly checked for completeness and consistency.

### Data processing and analyses

Data were coded, entered into Epi data v3.1 and transported to SPSS v23 for analysis. Bivariate binary logistic regression was conducted to see the association of each independent variable with the outcome variable. The candidate variables for multivariate logistic regression was set at p-value ≤ 0.25. The multivariable logistic regression models were fitted and adjusted odds ratios with 95% CI was calculated to identify the independent association with the outcome variable. P-value < 0.05 was considered statistically significant.

### Ethical consideration

The research and ethics review board of Jimma university, institute of health was approved this study, and the study was conducted in accordance with the principles of declaration of Helsinki. A letter of permission to proceed was obtained from the Jimma zone health department and relevant woreda health bureau. The study was carried out after written informed consent was obtained from each participant and each participant were informed about the aims of the study and the right to withdraw from the interview at any time. Information taken from the respondent was kept confidential at all levels of the study.

## Results

### Socio-demographic characteristics of the study participants

A total of 410 participant were enrolled in the study with an overall response rate of 99.5%. Two hundred eleven (51.5%) were males. The median age of the respondent was 28.00(IQR = 16) year. More than half of the study participant (62.7%) were Muslim and urban residents (52.2%) **(**Table 1).

**Table 1:** Socio-demographic characteristic of patient with PTB in Jimma zone, southwest Ethiopia, 2019 (n = 410).

Variables	Category	Frequency (N)	Percent (%)
**Sex**	Male	211	51.5
	Female	199	48.5
**Age in years**	18–24	132	32.2
	25–34	145	35.4
	35–44	57	13.9
	≥ 45	76	18.5
**Marital status**	Single	149	36.3
	Married	215	52.4
	Divorced	31	7.6
	Widowed	15	3.7
**Place of residence**	Rural	196	47.8
	Urban	214	52.2
**Religion**	Muslim	257	62.7
	Orthodox	113	27.6
	Protestant	40	9.8
**Educational status**	No formal education	90	22.00
	Primary (1–8)	147	35.9
	Secondary(9–12)	109	26.6
	College and above	64	15.6
**Occupation**	unemployed	128	31.2
	Government-employed	53	12.9
	Self-employed	229	55.9
**Family size**	≤5	358	87.3
	˃5	52	12.7

### Clinical and psychosocial characteristics of the study participant

Substantial number of the study participant (92.4%) were in the new TB treatment category. Over half (54.1%) were in intensive phase of treatment for TB. More than a half of the study particiupant (54.4%) reported high perceived stress. More than half (55.9%) were found to have depressive symptoms and reported current khat use (52.9%) (Table 2).

**Table 2:** Clinical and psychosocial factor distributions of patient with PTB, southwest Ethiopia, 2019 (n = 410).

Variables	Category	Frequency (N)	Percent (%)
**Phase of treatment**	intensive	222	54.1
	continuation	188	45.9
**Category of treatment**	new	379	92.4
	Return after default	10	2.4
	Relapse/retreatment	21	5.1
**Duration of illness**	≤ 1month	279	68.0
	˃ 1month	131	32.0
**HIV co-infection**	yes	86	21.0
	no	324	79.0
**family history of mental illness**	yes	87	21.2
	no	323	78.8
**BMI**	Underweight	182	44.4
	Not underweight	228	55.6
**Social support**	poor	171	41.7
	moderate	168	41.0
	Strong	71	17.3
**Depression**	Yes	229	55.9
	No	181	44.1
**Perceived stress level**	Low to average	187	45.6
	High	223	54.4
**Current Tobacco use**	No	389	94.9
	Yes	21	5.1
**Current Alcohol use**	Yes	90	22.0
	No	320	78.0
**Current cannabis use**	No	392	95.6
	Yes	18	4.4
**Current khat use**	No	193	47.1
	Yes	217	52.9

### Prevalence of perceived stigma among patient with PTB

The prevalence of perceived stigma among PTB patient was 57.1% (95% CI 52.2, 61.7). Perceived stigma was found to be higher among PTB patient who reported to have poor social support 136 (79.5%), high perceived stress 168 (75.3%), HIV co-infection 77(89.5%) and depressive symptoms 194 (84.7%) than their counterparts.

### Factors associated with perceived stigma among patients with PTB

Poor social support (AOR = 2.41: 95% CI: 1.06, 5.48), greater than a month duration of illness (AOR = 2.48: 95% CI: 1.33, 4.64, high perceived stress (AOR = 1.95; 95% CI:1.09, 3.49), and probable depression (AOR = 8.18: 95% CI: 4.40, 15.22) were significantly associated with perceived stigma.

Perceived stigma was about 2 times (AOR = 1.88; 95% CI: 1.05, 3.37) higher among current khat users than non-users. PTB patient who had HIV co-infection were 5.67 times (AOR = 5.67; 95% CI: 2.32, 13.87) more likely to have Perceived stigma than their counterparts. perceived stigma was observed to be higher among PTB patient who had HIV co-infection (89.5%) followed by patient screened positive for depression (84.7%). perceived stigma was reported less among PTB patient who reported to have poor social support(79.5%), high perceived stress (75.3%), greater than a month duration of illness (71.8%) and current khat use (65.0%) when compared to patient with HIV co-infection (AOR = 5.67 P< 0.001) and patient screened positive for depression (AOR = 8.18 P< 0.001) (Table 3).

**Table 3:** Factors associated with perceived stigma among patients with PTB, southwest Ethiopia, 2019 (n = 410).

Variable	Category	Perceived stigma		COR,95% (CI)	AOR,95% (CI)
		Yes N (%)	No N (%)		
**Sex**	Male	103(48.8)	108(51.2)	1	1
	Female	131(65.8)	68(34.2)	2.02 (1.35, 3.01)	1.58 (0.86, 2.93)
**Social support**	Poor	136(79.5)	35(20.5)	10.64 (5.58, 20.24)	2.41 (1.06, 5.48)*
	Moderate	79(47.0)	89(53.0)	2.43 (1.32, 4.45)	1.41 (0.66, 3.02)
	Strong	19(26.8)	52(73.2)	1	1
**Depression**	Yes	194(84.7)	35(15.3)	19.54(11.82,32.30)	8.18 (4.40, 15.22)**
	No	40(22.1)	141(77.9)	1	1
**Treatment Phase**	Intensive	168(75.7)	54(24.3)	5.75 (3.74, 8.83)	1.77 (0.98, 3.21)
	Continuation	66(35.1)	122(64.9)	1	1
**Duration of illness**	≤ 1 month	140(50.2)	139(49.8)	1	1
	˃ 1 month	94(71.8)	37(28.2)	2.52 (1.61,3.94)	2.48 (1.33, 4.64)*
**HIV co-infection**	Yes	77(89.5)	9(10.5)	9.10 (4.41, 18.77)	5.67 (2.32, 13.87)**
	No	157(48.5)	167(51.5)	1	1
**Perceived stress**	Low to average	66(35.3)	121(64.7)	1	1
	High	168(75.3)	55(24.7)	5.60 (3.65, 8.58)	1.95 (1.10, 3.49)*
**Current chat use**	Yes	141(65.0)	76(35.0)	1.99 (1.34, 2.97)	1.88 (1.05, 3.37)*
	No	93(48.2)	100(51.8)	1	1

Note:

* p<0.05

* * p< 0.001, 1 = reference category.

### Association among study variables

Evaluation of the association among data variables showed that duration of illness were observed to be associated with social support (χ2 = 8.39 P = 0.015), depression (χ2 = 12.13 P < 0.001) and perceived stress (χ2 = 5.72 P = 0.017). furthermore HIV co-infection were observed to have association with phase of treatment (χ2 = 19.06 P < 0.001), perceived social support (χ2 = 39.64 P <0.001), depression (χ2 = 27.49 P < 0.001) and perceived stress (χ2 = 9.60 P = 0.002) (S1 Table).

## Discussion

### Prevalence of perceived stigma among patient with PTB

The study revealed that a significant proportion of patient with PTB were found to have perceived stigma. This finding is in agreement with some of the previously conducted studies in Ethiopia [15, 21]. However, the finding was appeared to be higher than the study conducted in Walaita Sodo, Ethiopia [23]. The discrepancy might be attributed to the variations in instruments used and variation with the population studied. Unlike the previous study in which patient were included regardless of the TB type, the current study included only patient with PTB, which may account for the discrepancy. This is also supported by the finding from the previous study that patient with PTB were found to have high perceived stigma compared to patient with extra- PTB [23].

In agreement with the previous study conducted in Ethiopia [23], poor social support was significantly associated with perceived stigma. This might be related to the fact that poor social support tends to make patient to have a feeling of being neglected, isolated, worthless, and other various psycho-social consequences [28, 29].

It was found that the duration of illness more than a month was 2.48 times more likely to be associated with perceived stigma. This may be explained in part, by the fact that patient may hide their signs and symptoms because of fear of social isolation and the perception that the disease may be less severe [21]. The subsequent delay in diagnosis and initiation of the treatment, in turn intensify the perceived stigma.

The study demonstrated that patient with PTB who reported to have probable depression were about 8 times more likely to have perceived stigma. A similar finding was reported from other study conducted in Ethiopia [15]. People with mental illness like depression are subject to perceived stigma, having perceived stigma could also be a determinant factor for depression [30, 31].

Furthermore, HIV co-infection had a significant association with perceived stigma. Studies have been revealed that both TB and HIV are highly stigmatized disease, having these two together doubles the burden on the patient and contributed to high perceived stigma [7, 15, 32].

The study also revealed that current khat use had a significant association with perceived stigma. The finding is in line with the previous study which found that current use of substances such as khat and cigarette had an association with stigma [23].

Moreover, high perceived stress was significantly associated with perceived stigma. This may be explained in part, that having high perceived stress might contribute to negative coping behaviors such as substance use and isolation from society which, in turn, may further exacerbate the level of perceived stigma [33].

Although being unemployed and no formal education were not observed to have significant association with perceived stigma, the result showed that considerable number of participant were unemployed (31.2%) and had no formal education (22%).

No formal or low level of education, and being unemployed were shown to have relationship, that low level of education may prevent an individual from accessing most professional jobs and contributes to persistently low resources. Furthermore both could lead to economic vulnerability and social disadvantage, and hence increases the risk of getting the disease and create a negative impact on the patient as well as the wider public [34, 35]. Moreover previously conducted studies reported a significant association between low income and perceived stigma [36].

This study has noteable strengths. The study used a largely representative sample from multiple sites, making the findings potentially genearaliziable in a similar setting. Unlike the previously conducted studies in the country which included only limited variables, this study explored a wide range of confounding variables including the perceived level of stress, which is believed to be one of the potential confounders. The use of standard tools for data collection was also another strength of this study. This study was also not without limitations. As the data were collected through face-to-face interviews, there may be a social desirability bias.

Further, although huge efforts were made to include all potential confounders, yet, some variables such as the perceived severity of TB symptoms and side effects of medications were not covered in this study.

## Conclusion

More than a half of the study participant were found to have perceived stigma. Poor social support, high perceived stress, duration of illness more than a month, presence of depression, HIV co-infection, and current khat use were significantly associated with perceived stigma. Stigma reduction interventions such as community health education or counseling service are needed to lower patient-perceived TB stigma and the level of distress associated with it.

## Supporting information

S1 Table Association between data variables among patient with PTB, 2019 (n = 410). (DOCX)
