# Supplementary material for: Perceived stigma among patient with pulmonary tuberculosis at public health facilities in southwest Ethiopia: A cross-sectional study
Source: PLoS One. 2020 Dec 8;15(12):e0243433. doi: 10.1371/journal.pone.0243433 (PMC7731994; doi:10.1371/journal.pone.0243433)
Supplement: S1 Table — (DOCX) [file pone.0243433.s001.docx]

|  | | **Social support** | | | **Perceived stress** | | **Depression** | | **HIV co-infection** | | **Duration of illness** | |
| --- | --- | --- | --- | --- | --- | --- | --- | --- | --- | --- | --- | --- |
|  |  | poor | moderate | strong | Low to average | high | No | Yes | Yes | No | ≤ 1month | ˃1 month |
| **Sex** | Male | 78(37.0) | 89(42.2) | 44(20.9) | 113(53.6) | 98(46.4) | 114(54.0) | 97(46.0) | 36(41.9) | 175(54.0) | 153(54.8) | 58(44.3) |
|  | Female | 93(46.7) | 79(39.7) | 27(13.6) | 74(37.2) | 125(62.8) | 67(33.7) | 132(66.3) | 50(58.1) | 149(46.0) | 126(45.2) | 73(55.7) |
|  |  | ꭓ2 = 5.63 P= 0.06 | | | ꭓ2=10.41 P= 0.001 | | ꭓ2=16.40 P< 0.001 | | ꭓ2=3.54 P = 0.06 | | ꭓ2=3.57 P = 0.059 | |
| **Perceived Stress** | Low | 52(30.4) | 88(52.4) | 47(66.2) |  |  |  |  |  |  |  |  |
|  | High | 119(69.6) | 80(47.6) | 24(33.8) |  |  |  |  |  |  |  |  |
|  |  | ꭓ2=31.16 P < 0.001 | | |  | |  | |  | |  | |
| **Depression** | No | 36(21.1) | 89(53.0) | 56(78.9) | 130(69.5) | 57(30.5) |  |  |  |  |  |  |
|  | Yes | 135(78.9) | 79(47.0) | 15(21.1) | 51(22.9) | 172(77.1) |  |  |  |  |  |  |
|  |  | ꭓ2= 77 P< 0.001 | | | ꭓ2=87.88 P< 0.001 | |  | |  | |  | |
| **Current Khat use** | No | 71(41.5) | 85(50.6) | 37(52.1) | 101(54.0) | 92(41.3) | 98(54.1) | 95(41.5) | 42(48.8) | 151(46.6) | 126(45.2) | 67(51.1) |
|  | Yes | 100(58.5) | 83(49.4) | 34(47.9) | 86(46.0) | 131(58.7) | 83(45.9) | 134(58.5) | 44(51.2) | 173(53.4) | 153(54.8) | 64(48.9) |
|  |  | ꭓ2=3.67 P= 0.159 | | | ꭓ2=6.14 P= 0.013 | | ꭓ2=6.00 P= 0.014 | | ꭓ2=0.06 P = 0.80 | | ꭓ2=1.05 P= 0.30 | |
| **HIV co-infection** | Yes | 61(35.7) | 21(12.5) | 4(5.6) | 26(13.9) | 60(26.9) | 16(8.8) | 70(30.6) |  |  |  |  |
|  | No | 110(64.3) | 147(87.5) | 67(94.4) | 161(86.1) | 163(73.1) | 165(91.2) | 159(69.4) |  |  |  |  |
|  |  | ꭓ2= 39.64 P < 0.001 | | | ꭓ2=9.60 P = 0.002 | | ꭓ2= 27.49 P < 0.001 | |  | |  | |
| **Duration of illness** | ≤1 month | 103(36.9) | 125(44.8) | 51(18.3) | 139(49.8) | 140(50.2) | 140(50.2) | 139(49.8) | 58(20.8) | 221(79.2) |  |  |
|  | ˃ 1 month | 68(51.9) | 43(32.8) | 20(15.3) | 48(36.6) | 83(63.4) | 41(31.3) | 90(68.7) | 28(21.4) | 103(78.6) |  |  |
|  |  | ꭓ2=8.39 P= 0.015 | | | ꭓ2=5.72 P= 0.017 | | ꭓ2=12.13 P < 0.001 | | ꭓ2= 0 P= 0.99 | |  | |
| **Treatment phase** | intensive | 124(55.9) | 72(32.4) | 26(11.7) | 69(31.1) | 153(68.9) | 50(22.5) | 172(77.5) | 65(29.3) | 157(70.7) | 140(63.1) | 82(36.9) |
|  | Continua  tion | 47(25.0) | 96(51.1) | 45(23.9) | 118(62.8) | 70(37.2) | 131(69.7) | 57(30.3) | 21(11.2) | 167(88.8) | 139(73.9) | 49(26.1) |
|  |  | ꭓ2= 40.60 P < 0.001 | | | ꭓ2=39.92 P < 0.001 | | ꭓ2= 89.90 P < 0.001 | | ꭓ2= 19.06 P < 0.001 | | ꭓ2= 5.04 P = 0.025 | |

S1 Table Association among study variables
